# Gelation-driven Dynamic Systemic Resolution: *in situ* Generation and Self-Selection of an Organogelator

**DOI:** 10.1038/srep11065

**Published:** 2015-06-16

**Authors:** Lei Hu, Yang Zhang, Olof Ramström

**Affiliations:** 1 KTH – Royal Institute of Technology, Department of Chemistry, Teknikringen 30, Stockholm, Sweden

## Abstract

An organogelator was produced and identified from a dynamic imine system, resolved and amplified by selective gelation. The formation of the organogel was monitored *in situ* by ^1^H NMR, showing the existence of multiple reversible reactions operating simultaneously, and the redistribution of the involved species during gelation. The formed organogelator proved effective with a range of organic solvents, including DMSO, toluene, and longer, linear alcohols.

Dynamic chemistry enabling the change of constitution of molecular entities has proven to be one of the most efficient methods for the generation and exploration of complex systems^1^,^2^. Such systems for example possess adaptive features, responsive to the system environment, and may undergo self-correcting and self-selection processes to establish optimal conditions. The adaptive nature of the dynamic systems thus forces its constituents to constantly re-equilibrate in the presence of internal or external pressures, resulting in the amplification of the optimal constituents at the expense of all others. A wide range of applications using this chemistry has also been demonstrated^3^,^4^,^5^,^6^,^7^,^8^,^9^,^10^,^11^,^12^,^13^,^14^,^15^, including selection and identification of, for example functional materials^16^,^17^,^18^ and various host- or guest entities^19^,^20^.

Kinetically controlled processes coupled to dynamic systems lead to the concept of dynamic systemic resolution (DSR), in principle enabling kinetic resolution of selected constituents^21^,^22^. An attractive feature of DSR is the possibility of acquiring complete amplification of the optimal species, regardless of the specific distributions formed in the thermodynamic equilibria, provided the kinetic steps are selective. We have for example shown these features in enzyme-catalyzed selection and asymmetric synthesis of different structures^23^,^24^,^25^,^26^,^27^,^28^,^29^, and reported that selective crystallization can be used to control and drive dynamic systems to the amplification of specific constituents^30^,^31^. In the present study, we expand the concept of DSR to the field of gelation, in which an organic liquid is entrapped in a solid three-dimensional network by capillary forces and adhesion^32^. Owing to its unique physical properties, increasing attention has been continuously paid to the development of novel organogels, and successful applications have been achieved in fields such as pharmaceutics, cosmetics, food processing, as well as in the oil industry^33^,^34^. Even though supramolecular hydrogel-based gelation has been used as driving force for component selection^35^,^36^, the study of organogels remains unexplored. Herein, we report the first example of *in situ* generation and self-selection of an efficient organogelator at the dynamic constitutional level (Fig. 1).

## Results and discussion

**Figure 2:**
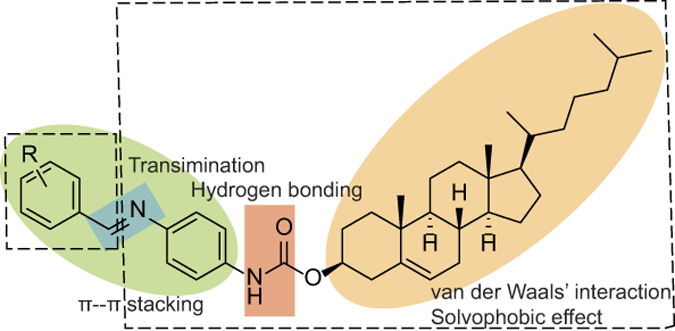


In general, a range of intermolecular interactions and effects between the individual organogelator structures and the solvent, *e.g.*, hydrogen bonding, π- π stacking, solvophobic effects, and van der Waals’ interactions, contribute to the formation of the 3D gel network. In the present case, the following design criteria of the potential organogelators were devised (Fig. 2): an imine element to introduce dynamic properties of the entities and extending the conjugated part of the system; an aromatic unit to admit potential π-π stacking interactions; a carbamate element to enable intermolecular hydrogen bonds; and a solvophobic moiety to result in solvent-dependent aggregation. Consequently, the dynamic C = N bonds were synthesized from aromatic aldehydes and aromatic amine units, allowing the continuous formation of all possible products under imine exchange conditions. Cholesterol and its derivatives have been commonly used for the synthesis of organogelators due to the strongly lipophilic character of the steroid motif, which tends to self-assemble in polar solvents^37^,^38^. Therefore, the cholesterol core was also chosen as an integral part of the dynamic system, linked to the remaining structure through a carbamate moiety.

**Figure 3:**
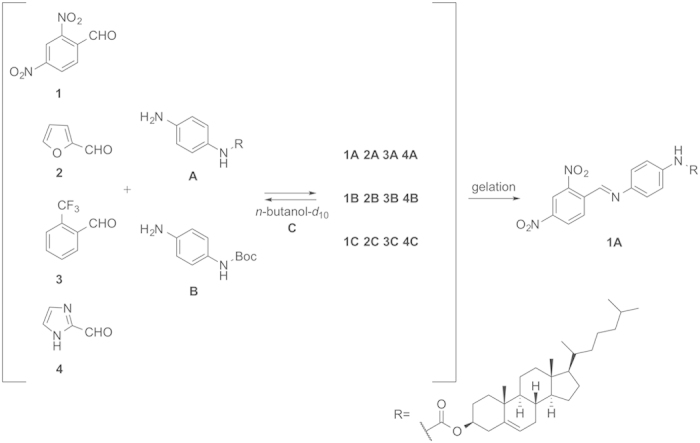


**Figure 4:**
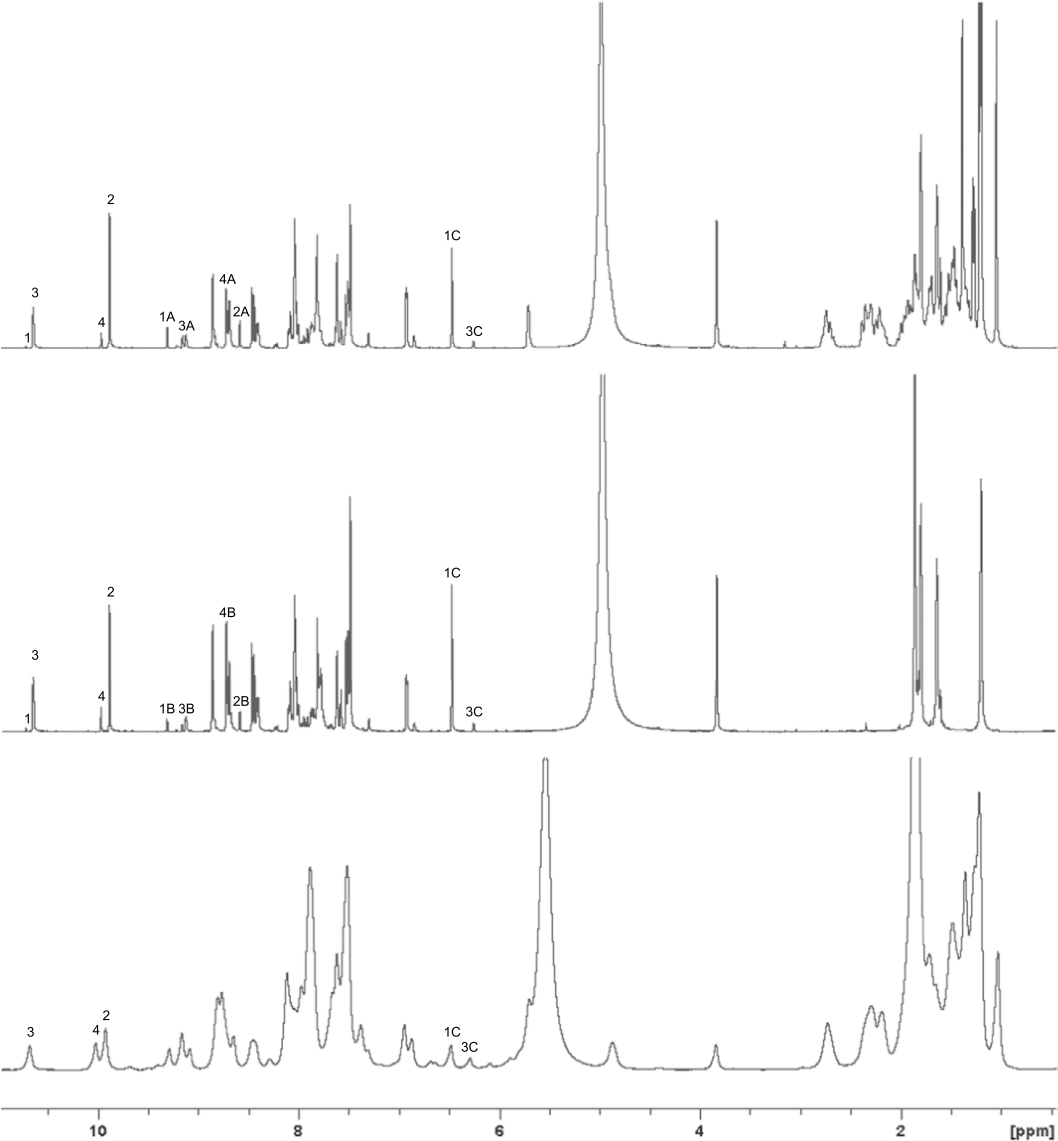
^1^H NMR spectra of dynamic imine system: **a**) equilibrium between aldehydes and amine **A**; **b**) equilibrium between aldehydes and amine **B**; **c**) constituent distribution after gelation.

Based on these criteria, four aromatic aldehydes (**1–4**), representing both five- and six-membered rings, and two amines, either with a carbamate-linked cholesterol core (**A**) or using a *tert*-butyloxycarbonyl (Boc) group (**B**), were selected as components. Prototype dynamic systems were subsequently generated in *n*-butanol-*d*_10_ with each component present in equimolar amount (Fig. 3). The equilibration processes between the aldehydes and each of the amines were examined separately due to the overlap of the product signals (Fig. 4). Imine formation and exchange occurred rapidly, and according to the ^1^H NMR analyses, both equilibria were attained within one hour. In the system between the aldehydes and amine **A**, the product ratio between imines **1A**, **2A**, **3A** and **4A** was 1:1.6:1.8:2.5 compared to 1:2.1:2.9:9.0 in the equilibrium between the aldehydes and amine **B**. In the first system, CDCl_3_ was added (20% v/v) to prevent precipitation, whereas this was unnecessary in the second system. Interestingly, even though the substitution pattern of the aromatic ring of aldehyde **1** involves two very electron-withdrawing nitro groups, the corresponding product **1A** was the least preferred compound compared with the other imine products. However, this effect proved to be due to the favored formation of hemiacetal **1C** from the addition of deuterated *n*-butanol to aldehyde **1**. In the three parallel reversible reactions, the hemiacetal formation dominated the competition. Small amounts of hemiacetal **3C** were also detected in both equilibria, while furfural **2** and imidazole-2-carboxaldehyde **4** showed a tendency to form mainly imine products.

Following the evaluation of the dynamic features, a complete dynamic system with all six starting materials was generated in *n*-butanol-*d*_10_ in an NMR tube. The resulting mixture was heated until the solution became transparent and then allowed to cool down to room temperature, at which time an organogel was obtained. According to the ^1^H NMR spectrum, almost all of the hemiacetal **1C** and most of the cholesterol peaks disappeared with very weak formation of the imine **1B** (Fig. 4c). Meanwhile, the other three aldehydes and Boc-containing species were obvious. This result indicated the possible amplification of organogelator **1A** from the dynamic system as its signal and the related components signals were very weak in the ^1^H NMR spectrum due to the solidification. To further support these results, the organogel was filtered and the residue was washed with cold deuterated *n*-butanol-*d*_10_. The solid and the filtrate were analyzed respectively, showing that the filtrate ^1^H NMR spectrum was identical to that obtained after gelation, and the solid was analytically pure imine **1A** (84% yield). The gelation process was therefore efficient as the driving force for the re-equilibration of the dynamic system, leading to the exclusive formation of product **1A**. The formation furthermore occurred at the expense of the hemiacetals and the other imines, since the selected constituent belonged to one of the most unfavored species in the original equilibrium. Control experiments were also conducted showing that among all the starting materials and possible products imine **4A** could also gel *n*-butanol in half an hour. Considering that imine **4A** is not only the most dominant species in the first equilibrium but also possesses gelation property, the better gelating efficiency of imine **1A** is crucial to the successful resolution of the dynamic system.

To further investigate the gelation property of compound **1A**, 17 solvents were tested at a gelator concentration of 3 w/v% (Table 1). Of the solvents, gelation was observed in DMSO and toluene, as well as in aliphatic alcohols from *n*-butanol to *n*-octanol. All other solvents, including shorter *n*-alcohols and branched alcohols resulted in solution or precipitation, indicating the gelation preference for the longer, linear alcohol structures.

**Figure 5:**
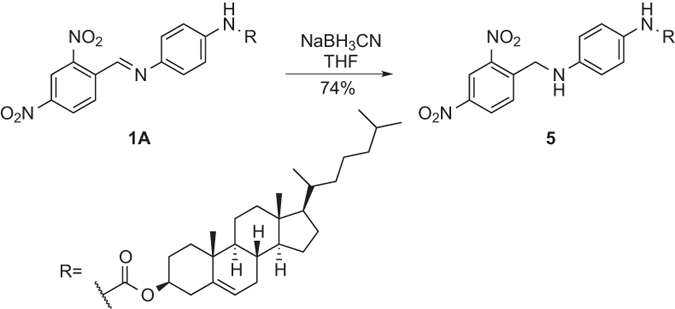


To investigate the impact of the imine bond on its gelation property, the C = N double bond of compound **1A** was reduced (Fig. 5). A weak reducing agent NaBH_3_CN, which is specifically used for reductive amination, was applied and resulted in smooth production of compound **5** in 74% yield. The gelation behavior of reduced product **5** was assessed using the same solvents. No gel was formed in any of those solvents even with increased gelator concentration, indicating the importance of the C = N bond as a key factor for keeping good gelation properties. The rationale behind is probably due to the increased degree of rotation with the formation of C-N single bond as well as the loss of conjugation, both perturbing the assembly of the gelator network.

In summary, we have successfully designed and established a dynamic system consisting of multiple organogelator candidates, which were subsequently resolved in an *in situ* gelation process by which the optimal constituent was amplified and identified. The whole process could be monitored by ^1^H NMR spectroscopy, which following the resolution indicated the selective formation of a cholesterol-containing imine product produced in high yield. This method represents a potent approach to rapid generation and identification of organogelators at the constitutional level, enabling the development of novel organogelators.

## Methods

### General

All commercially available starting materials were of reagent grade and used as received. ^1^H NMR and ^13^C NMR data were recorded on a Bruker Avance DMX 500 at 500 (125) MHz and/or a Bruker Avance 400 (100) MHz, respectively. Chemical shifts are reported as δ values (ppm) with CDCl_3_ (^1^H NMR δ 7.26, ^13^C NMR δ 77.16) as internal standard. *J* values are given in Hertz (Hz). Thin layer chromatography (TLC) was performed on precoated Cromatofolios AL Silica gel 60 F_254_ (Merck, Darmstadt, Germany), visualized with UV-detection. Flash column chromatography was performed on silica gel 60, 0.040–0.063 mm (SDS). High-resolution mass spectra (HRMS) were analyzed by Proteoomika tuumiklabor, TÜ Tehnoloogiainstituut, Tartu, Estonia.

### Dynamic systemic resolution

1 equiv. of each compound **1**, **2**, **3**, **4**, **A**, and **B** (0.06 mmol, 0.15 M) was added to an NMR tube, and then *n*-butanol-*d*_10_ (0.4 mL) was added. The mixture was heated until a transparent solution was obtained, and cooled to room temperature. The gel was filtered, and the solid was washed with cold *n*-butanol-*d*_10_ (0.2 mL). The concentrated filtrate and the solid residue were analyzed by ^1^H NMR.

## Additional Information

**How to cite this article**: Hu, L. *et al.* Gelation-driven Dynamic Systemic Resolution: *in situ* Generation and Self-Selection of an Organogelator. *Sci. Rep.* **5**, 11065; doi: 10.1038/srep11065 (2015).

## Supplementary Material

Supplementary Information

## Figures and Tables

**Figure 1:**
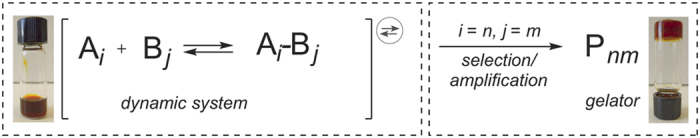
Concept of gelation-driven selection and amplification of organogelator from a thermodynamically controlled dynamic system. A series of substrates P_*ij*_ is dynamically formed from individual components A_*i*_and B_*j*_, and the dynamic system is subsequently resolved *via* gel formation of product P_*nm*_.

**Table 1:** Gelation screening.

**Gelation**	**Solution**	**Precipitation**
DMSO	dioxane	acetonitrile
toluene	DMF	ethanol
*n*-butanol	THF	*i*-propanol
*n*-pentanol	*n*-propanol	*t*-butanol
*n*-hexanol	*i*-butanol	1,2-dichlorobenzene
*n*-heptanol		
*n*-octanol		
