# Supplementary material for: Gelation-driven Dynamic Systemic Resolution: in situ Generation and Self-Selection of an Organogelator
Source: Sci Rep. 2015 Jun 16;5:11065. doi: 10.1038/srep11065 (PMC4462186; doi:10.1038/srep11065)
Supplement: Supplementary Information [file srep11065-s1.pdf]

SUPPORTING INFORMATION

Gelation-driven Dynamic Systemic Resolution: *in situ* Generation and Self-Selection of an Organogelator

*Lei Hu, Yang Zhang and Olof Ramström**

KTH - Royal Institute of Technology, Department of Chemistry,
Teknikringen 30, S-10044 Stockholm, Sweden; Email: ramstrom@kth.se

Table of contents

Dynamic systemic resolution 1

Synthesis 1
tert-butyl 4-aminophenylcarbamate (**B**) 1
(10*R*,13*R*,17*R*)-10,13-dimethyl-17-((*R*)-6-methylheptan-2-yl)-2,3,4,7,8,9,10,11,12,13,14,15,16,17-tetradecahydro-1*H*-cyclopenta[a]phenanthren-3-yl 4-aminophenylcarbamate (**A**) 2
(3*S*,8*S*,9*S*,10*R*,13*R*,14*S*,17*R*)-10,13-dimethyl-17-((*R*)-6-methylheptan-2-yl)-2,3,4,7,8,9,10,11,12,13,14, 15,16,17-tetradecahydro-1*H*-cyclopenta[a]phenanthren-3-yl (4-(((*E*)-2,4-dinitrobenzylidene)amino) phenyl)carbamate (**1A**) 2
(3S,8S,9S,10R,13R,14S,17R)-10,13-dimethyl-17-((R)-6-methylheptan-2-yl)-2,3,4,7,8,9,10,11,12,13,14,15,16,17-tetradecahydro-1H-cyclopenta[a]phenanthren-3-yl (4-(((E)-(1H-imidazol-2-yl)methylene)amino)phenyl)carbamate (**4A**) 3
(3S,8S,9S,10R,13R,14S,17R)-10,13-dimethyl-17-((R)-6-methylheptan-2-yl)-2,3,4,7,8,9,10,11,12,13,14,15,16,17-tetradecahydro-1H-cyclopenta[a]phenanthren-3-yl 4-(2,4-dinitrobenzylamino)phenylcarbamate (**5**) 3

NMR spectra 4
Figure S 1. ^{1}H NMR and ^{13}C NMR spectra of compound **B** 4
Figure S 2. ^{1}H NMR and ^{13}C NMR spectra of compound **A** 5
Figure S 3. ^{1}H NMR and ^{13}C NMR spectra of compound **1A** 6
Figure S 4. ^{1}H NMR and ^{13}C NMR spectra of compound **4A** 7
Figure S 5. ^{1}H NMR and ^{13}C NMR spectra of compound **5** 8

Dynamic systemic resolution

1 equiv. of each compound 1, 2, 3, 4, A, and B (0.06 mmol, 0.15 M) was added to an NMR tube, and then *n*-butanol-d_{10} (0.4 mL) was added. The mixture was heated until a transparent solution was obtained, and subsequently cooled to room temperature**.** The gel was filtered, and the solid was washed with cold *n*-butanol-d_{10} (0.2 mL). The concentrated filtrate and the solid residue were analyzed by ^{1}H NMR.

Synthesis

tert-butyl 4-aminophenylcarbamate (B)

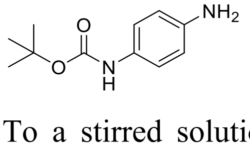

To a stirred solution of *p*-phenylenediamine (594 mg, 5.5 mmol) in dichloromethane (10 mL), cooled in an ice-bath, was added a solution of di-*tert*-butyl dicarbonate (720 mg, 3 mmol) in dichloromethane (5 mL) dropwise. The solution was stirred at room temperature for 4 h and then concentrated under reduced pressure. The crude product was purified by column chromatography

(hexane/ethyl acetate = 2:1, v:v) to yield a white solid **B** (588 mg, yield 91%). ^{1}H NMR (400 MHz, $CDCl_3$): δ 7.13 (d, 2H, *J* = 7.4Hz), 6.63 (d, 2H, *J* = 8.68Hz), 6.24 (s, 1H), 3.52(s, 2H), 1.50(s, 9H); ^{13}C NMR (125 MHz, $CDCl_3$): δ 153.53, 142.57, 129.92, 121.12, 115.72, 80.24, 28.55.

(10*R*,13*R*,17*R*)-10,13-dimethyl-17-((*R*)-6-methylheptan-2-yl)-2,3,4,7,8,9,10,11,12,13,14,15,16,17-tetradecahydro-1*H*-cyclopenta[a]phenanthren-3-yl 4-aminophenylcarbamate (A)

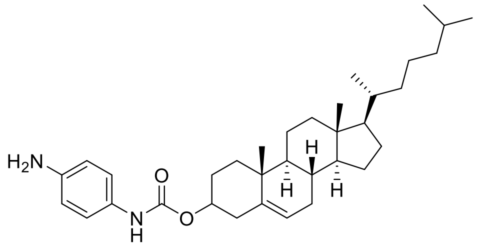

To a stirred solution of *p*-phenylenediamine (130 mg, 1.2 mmol) and pyridine (57 μL, 0.7 mmol) in dichloromethane (3 mL), cooled in an ice-bath, was added a solution of cholesteryl chloroformate (314 mg, 0.7 mmol) in dichloromethane (3 mL) dropwise. The solution was stirred at 0 °C for 4 h, diluted with dichloromethane (10 mL) and then washed with water, brine and dried over sodium sulfate. After concentrating the solution under reduced pressure, the crude was purified by column chromatography (hexane/ethyl acetate = 2:1) to get a white solid **A** (273 mg, yield 75%). ^{1}H NMR (500MHz, $CDCl_3$): δ 7.15(br, 2H), 6.66(d, 2H, *J* = 8.56 Hz), 6.32(s, 1H), 5.39(m, 1H), 4.59(m, 1H), 3.62(s, 2H), 0.68-2.43(m, 40H); ^{13}C NMR (125MHz, $CDCl_3$): δ 153.75, 142.74, 139.93, 129.62, 122.83, 121.03, 115.80, 74.77, 56.83, 56.29, 50.15, 42.46, 39.84, 39.67, 38.65, 37.13, 36.74, 36.33, 35.94, 32.04, 28.40, 28.27, 28.16, 24.47, 24.01, 23.04, 22.72.

(3*S*,8*S*,9*S*,10*R*,13*R*,14*S*,17*R*)-10,13-dimethyl-17-((*R*)-6-methylheptan-2-yl)-2,3,4,7,8,9,10,11,12,13,14, 15,16,17-tetradecahydro-1*H*-cyclopenta[a]phenanthren-3-yl (4-(((*E*)-2,4-dinitrobenzylidene)amino) phenyl)carbamate (1A)

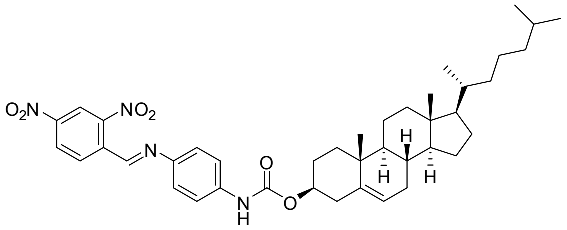

To a solution of compound **A** (40 mg, 0.0768 mmol) in anhydrous dichloromethane (1.6 mL) with 4Å molecular sieves, was added compound **1** (15 mg, 0.0768 mmol). The reaction was stirred under reflux for 4 h, and filtrated. The filtrate was concentrated under reduced pressure to yield compound **1A** as orange solid (51 mg, 95%). ^{1}H NMR (400MHz, $CDCl_3$): δ 9.03(s, 1H), 8.91(d, 1H, *J* = 1.82 Hz), 8.61(d, 1H, *J* = 8.63 Hz), 8.53 (dd, 1H, *J* = 8.70, 1.83 Hz), 7.49(d, 2H, *J* = 8.35 Hz), 7.36(d, 2H, *J* = 8.54 Hz), 6.68(s, 1H), 5.41(d, 1H, *J* = 3.93 Hz), 4.63 (m, 1H), 2.30-2.49 (m, 2H), 1.77-2.07 (m, 5H), 0.94-1.72 (m, 24H), 0.92(d, 3H, *J* = 6.50 Hz), 0.87 (dd, 6H, *J* = 6.68, 1.26 Hz), 0.68 (s, 3H); ^{13}C NMR (125MHz, $CDCl_3$): δ 152.83, 151.13, 148.98, 148.36, 144.87, 139.48, 138.51, 136.15, 131.27, 127.44, 122.94, 122.79, 120.41, 119.14, 75.30, 56.69, 56.13, 50.00, 42.33, 39.73, 39.53, 38.45, 36.97, 36.60, 36.19, 35.83, 31.93, 31.87, 28.26, 28.09, 28.05, 24.31, 23.85, 22.86, 22.60, 21.07, 19.37, 18.74, 11.89; HRMS (ESI-TOF): 699.4122 ($[M+1]^+$, $C_{41}H_{55}N_4O_6$; calc. 699.4116).

(3S,8S,9S,10R,13R,14S,17R)-10,13-dimethyl-17-((R)-6-methylheptan-2-yl)-2,3,4,7,8,9,10,11,12,13,14,15,16,17-tetradecahydro-1H-cyclopenta[a]phenanthren-3-yl (4-(((E)-(1H-imidazol-2-yl)methylene)amino)phenyl)carbamate (4A)

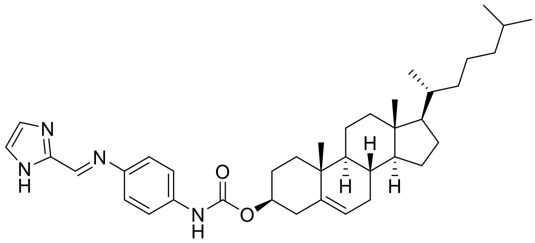

To a solution of compound **A** (32 mg, 0.06 mmol) in *n*-butanol (0.4 mL) was added compound **4** (6 mg, 0.06 mmol), and the mixture was heated in a cap-sealed vial until transparence. The solution was then cooled to room temperature and stood for 30 minutes, at which time a light grey gel was formed. The trapped solvent was died under N_2 flow and the residue was further dried under vacuum to afford pure compound **4A** (36 mg, quantitative yield). ^{1}H NMR (400MHz, $CDCl_3$): δ 8.47(s, 1H), 7.41(d, 1H, J = 8.80 Hz), 7.22(d, 2H, J = 8.71 Hz), 6.64 (s, 1H), 5.41(m, 1H), 4.62(m, 1H),3.66(t, 1H, J = 6.58 Hz), 2.30-2.49 (m, 2H), 1.77-2.07 (m, 5H), 0.89-1.70 (m, 27H), 0.87 (dd, 6H, J = 6.65, 1.59 Hz), 0.69 (s, 3H); ^{13}C NMR (125MHz, $CDCl_3$): δ 153.04, 148.09, 145.56, 145.22, 139.57, 137.16, 122.85, 121.91, 119.51, 75.17, 62.73, 56.72, 56.19, 50.04, 42.34, 39.76, 39.53, 38.47, 36.99, 36.60, 36.21, 35.81, 34.89, 31.91, 29.70, 28.23, 28.12, 28.01, 24.29, 23.86, 22.81, 22.55, 21.07, 19.34, 18.90, 18.73, 13.84, 11.87; HRMS (ESI-TOF): 599.4316 ($[M+1]^+$, $C_{38}H_{54}N_4O_2$; calc. 599.4320).

(3S,8S,9S,10R,13R,14S,17R)-10,13-dimethyl-17-((R)-6-methylheptan-2-yl)-2,3,4,7,8,9,10,11,12,13,14,15,16,17-tetradecahydro-1H-cyclopenta[a]phenanthren-3-yl 4-(2,4-dinitrobenzylamino)phenylcarbamate (5)

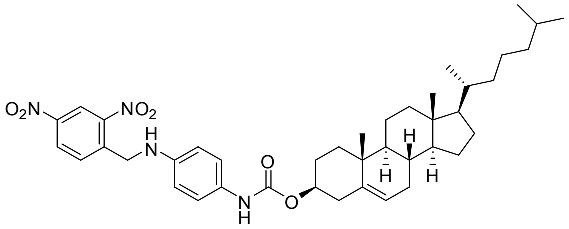

To a solution of compound **1A** (32 mg, 0.05 mmol) in anhydrous tetrahydrofuran (3 mL) was added $NaBH_3CN$ (32 mg, 0.5 mmol). The solution was stirred under reflux for 12 h, quenched by aqueous ammonium chloride solution (0.1 M, 10 mL) and then washed with saturated $NaHCO_3$, water and brine and dried over sodium sulfate. After concentrating the solution under reduced pressure, the crude was purified by column chromatography (hexane/ethyl acetate = 4:1) to get an orange solid **5** (23 mg, 55%). ^{1}H NMR (400MHz, $CDCl_3$): δ 8.94 (d, 1H, J = 2.46 Hz), 8.39 (dd, 1H, J_1= 8.72 Hz, J_2= 2.45 Hz), 7.93 (d, 1H, J = 8.56 Hz), 7.16 (d, 2H, J = 8.38 Hz), 6.46 (d, 2H, J = 8.48 Hz), 6.31 (s, 1H). 5.38 (s, 1H). 4.82 (d, 2H, J = 5.61 Hz), 4.57 (m, 1H), 4.31 (s, 1H), 2.31-2.43 (m, 2H), 1.85-2.02 (m, 5H), 0.92-1.60 (m, 24H), 0.91 (d, 3H, J = 6.35 Hz), 0.85 (dd, 6H, J_1= 6.51 Hz, J_2=1.56 Hz), 0.68 (s, 3H); ^{13}C NMR (100 MHz, $CDCl_3$): δ 148.06, 147.16, 143.18, 143.00, 139.77, 131.23, 129.79, 127.77, 122.84, 121.36, 120.90, 115.80, 113.45, 77.36, 56.84, 56.26, 50.19, 46.39, 42.44, 39.85, 39.67, 38.60, 37.15, 36.73, 36.31, 35.90, 32.00, 29.87, 28.42, 28.27, 28.16, 24.47, 24.01, 23.01, 22.70, 21.19, 19.53, 18.91, 11.95; HRMS (ESI-TOF): 701.4271 ($[M+1]^+$, $C_{41}H_{55}N_4O_6$; calc. 701.4233).

NMR spectra

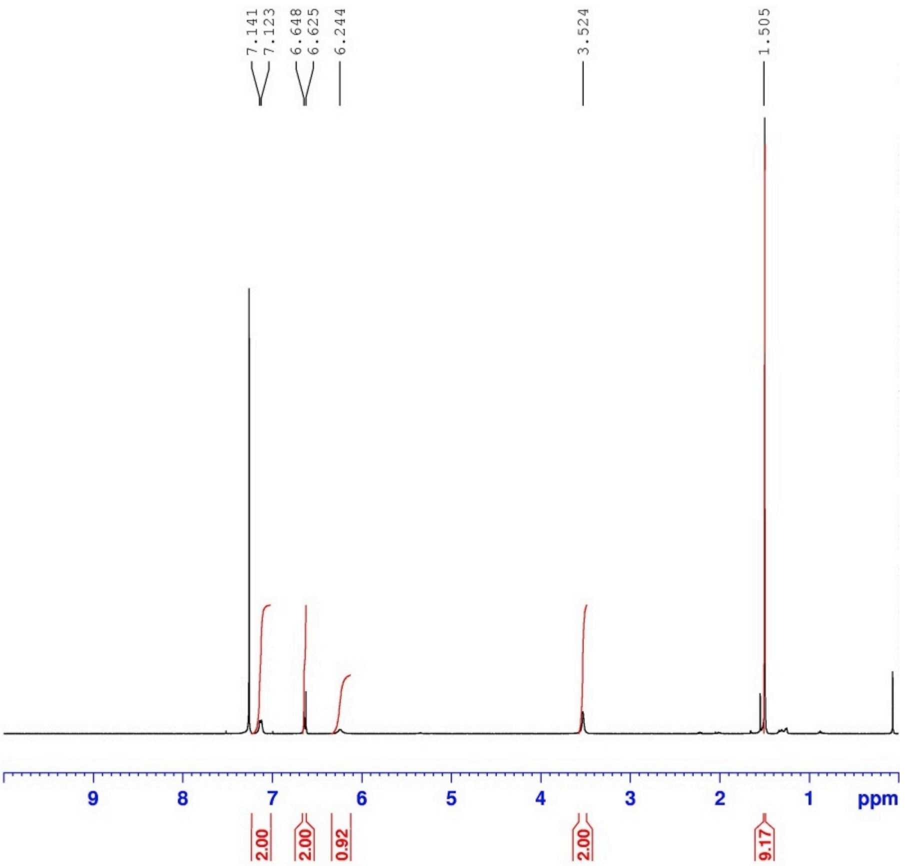

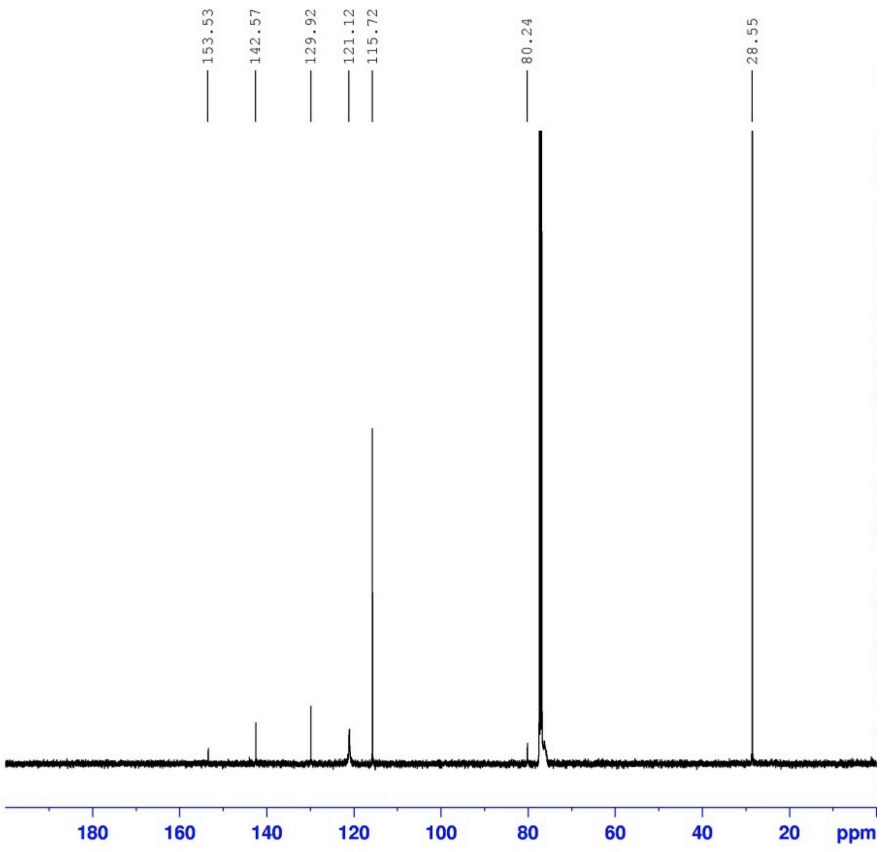

Figure S 1. ^{1}H NMR and ^{13}C NMR spectra of compound **B**

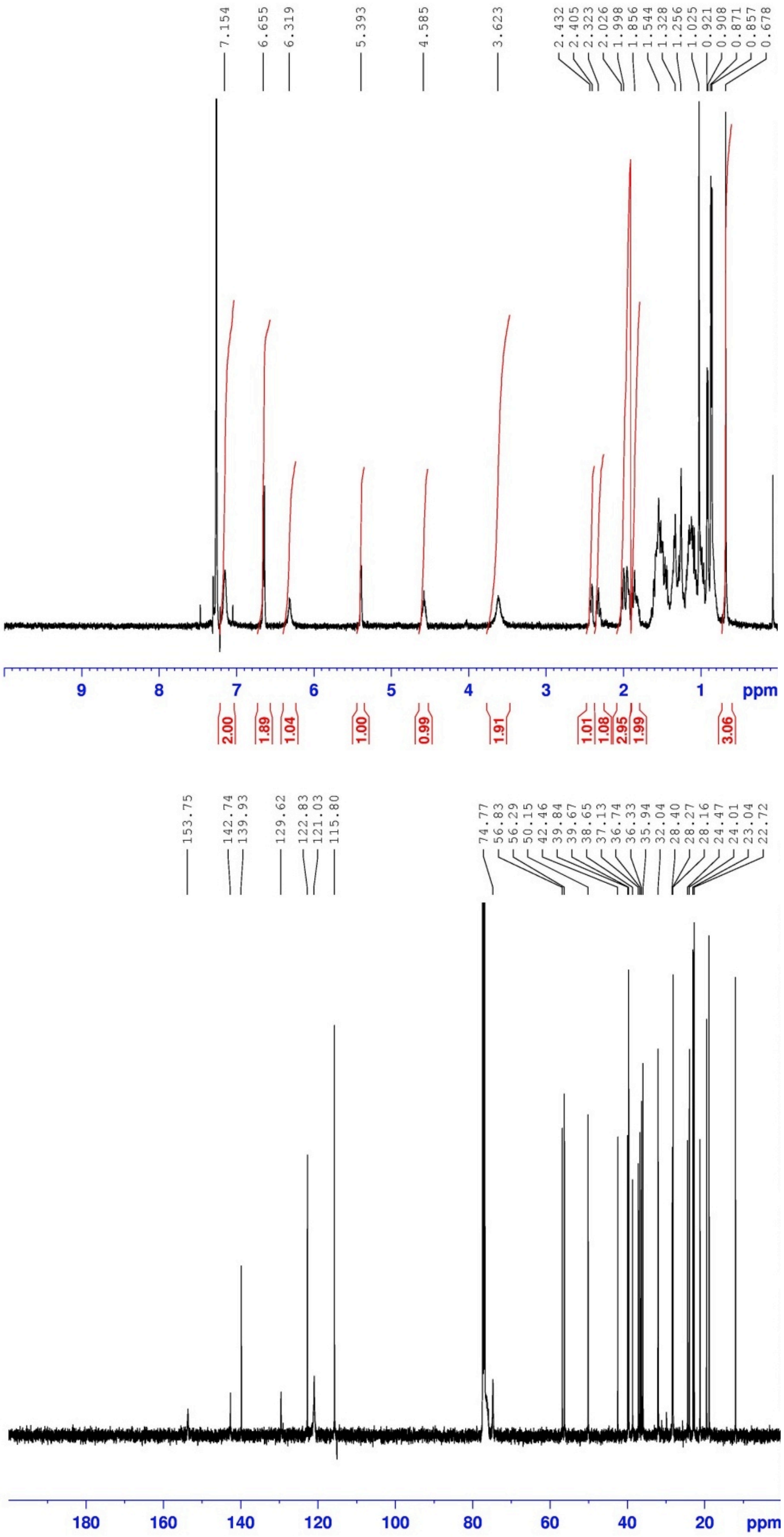

Figure S 2. ^{1}H NMR and ^{13}C NMR spectra of compound **A**

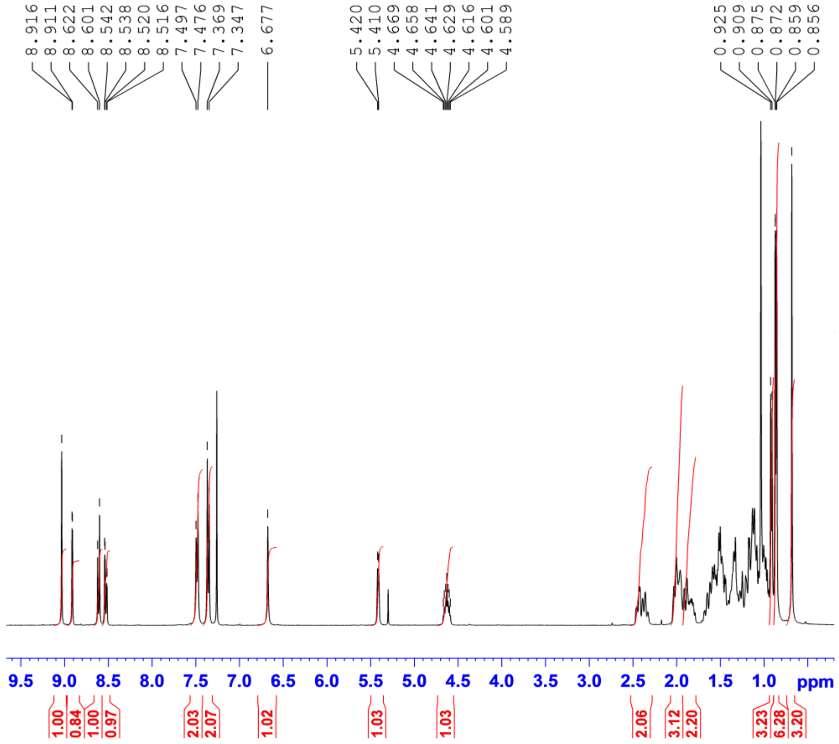

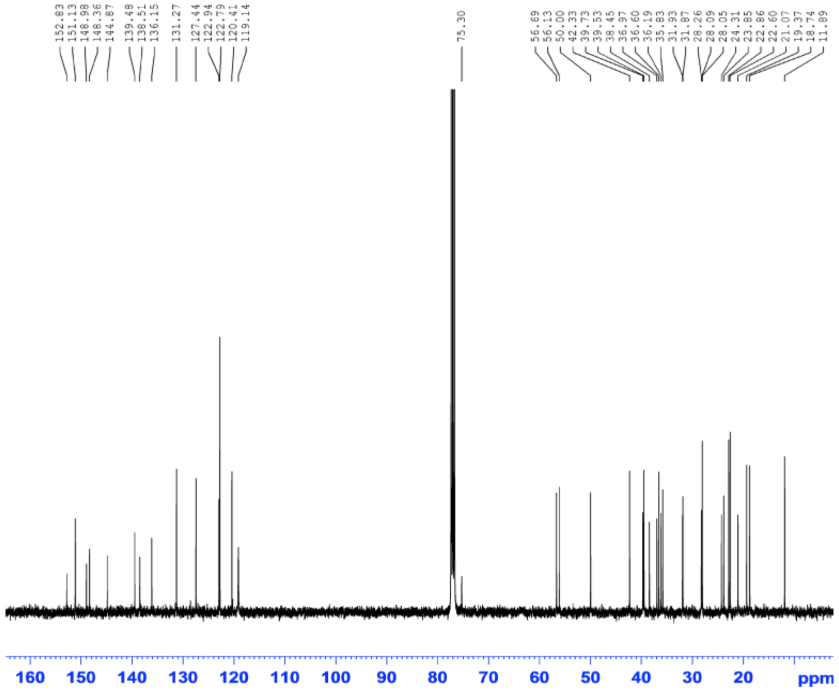

Figure S 3. ^{1}H NMR and ^{13}C NMR spectra of compound **1A**

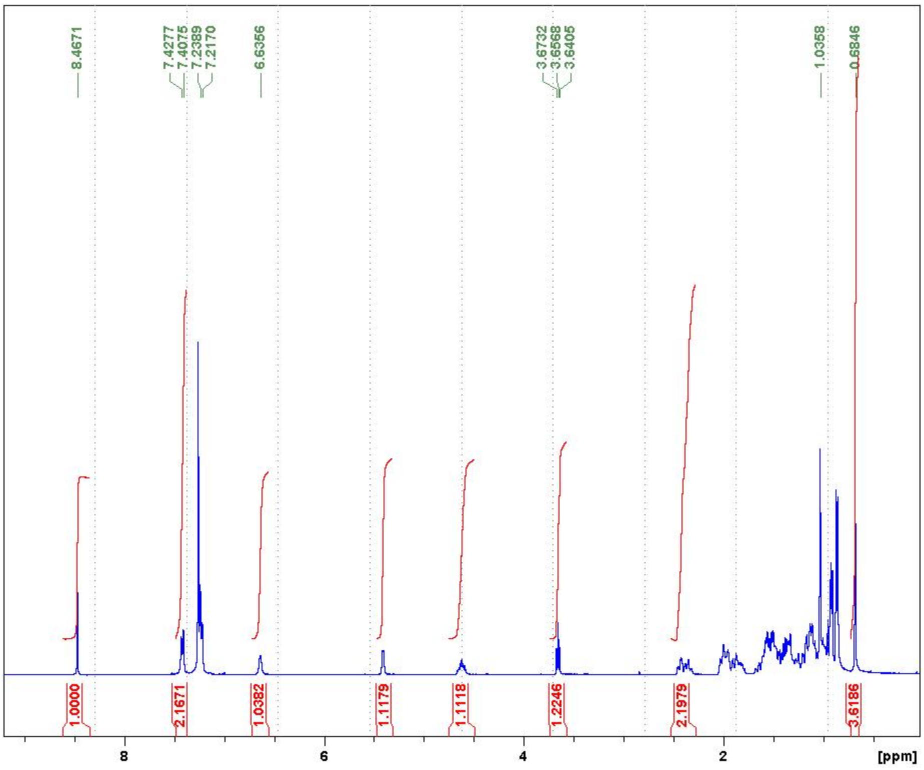

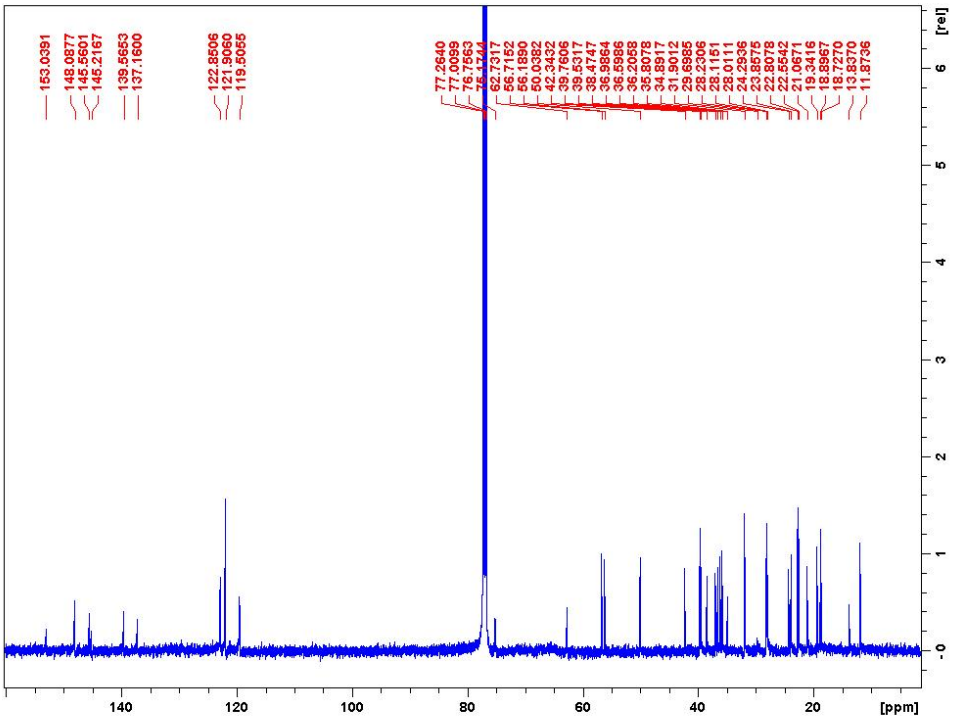

Figure S 4. ^{1}H NMR and ^{13}C NMR spectra of compound **4A**

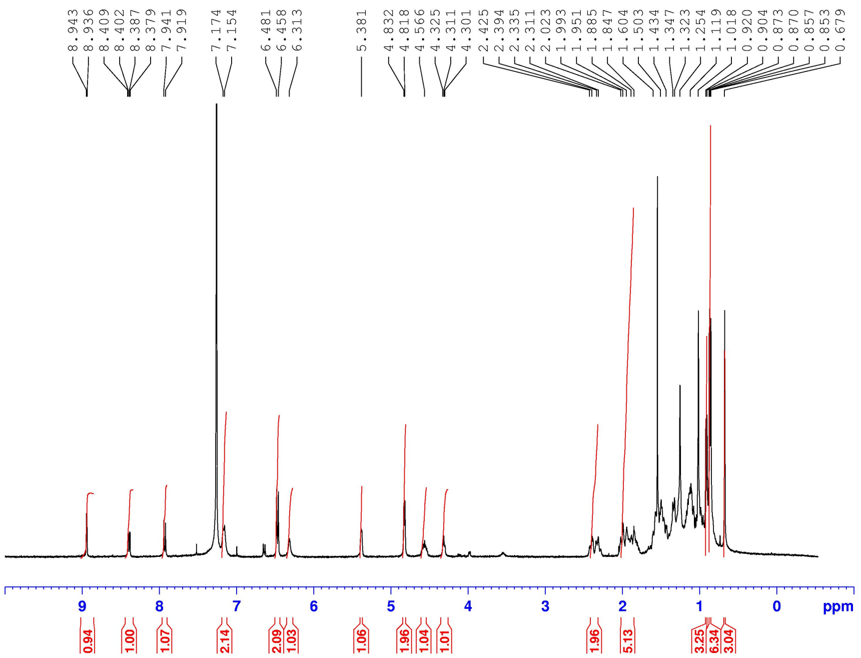

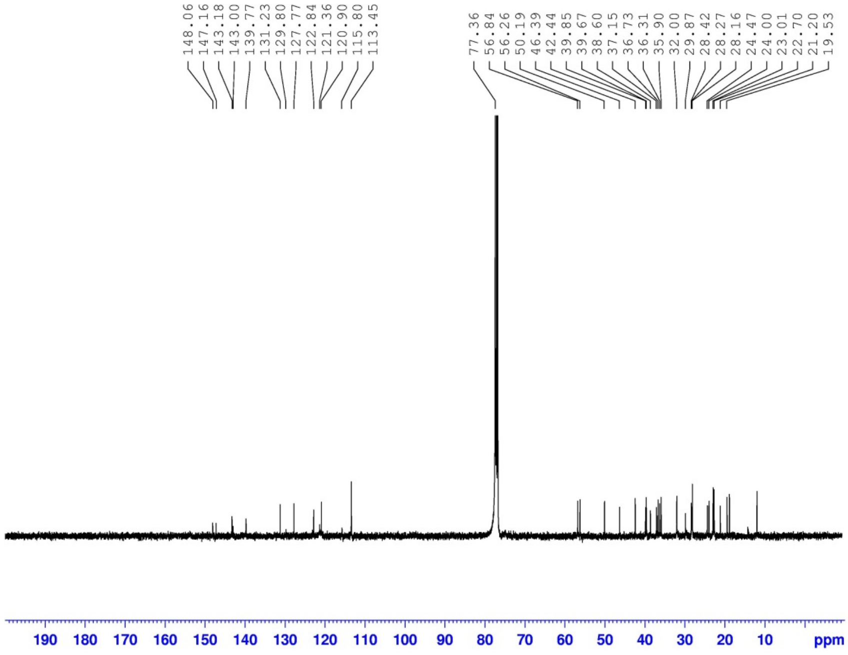

Figure S 5. ^{1}H NMR and ^{13}C NMR spectra of compound **5**

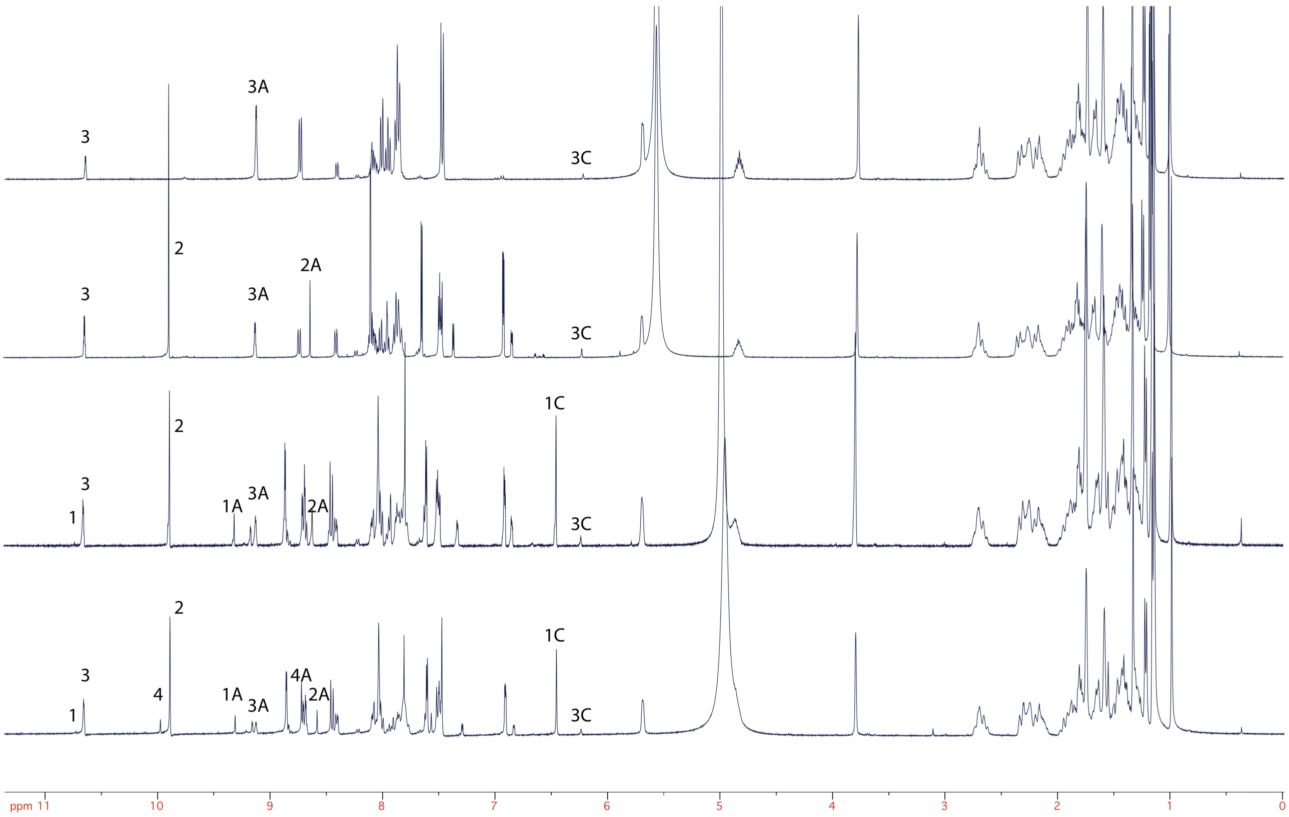

Figure S 6. ^{1}H NMR spectra of dynamic imine system **a**: 1) equilibrium of aldehyde **3**, amine **A** and deuterated *n*-butanol **C**; 2) equilibrium of aldehyde **2**, aldehyde **3**, amine **A** and deuterated *n*-butanol **C**; 3) equilibrium of aldehyde **1**, aldehyde **2**, aldehyde **3**, amine **A** and deuterated *n*-butanol **C**; 4) equilibrium of aldehyde **1**, aldehyde **2**, aldehyde **3**, aldehyde **4**, amine **A** and deuterated *n*-butanol **C**; ^{1}H NMR analyses were conducted with all building blocks of 40 mM concentration with 20% v/v $CDCl_3$ in deuterated *n*-butanol.

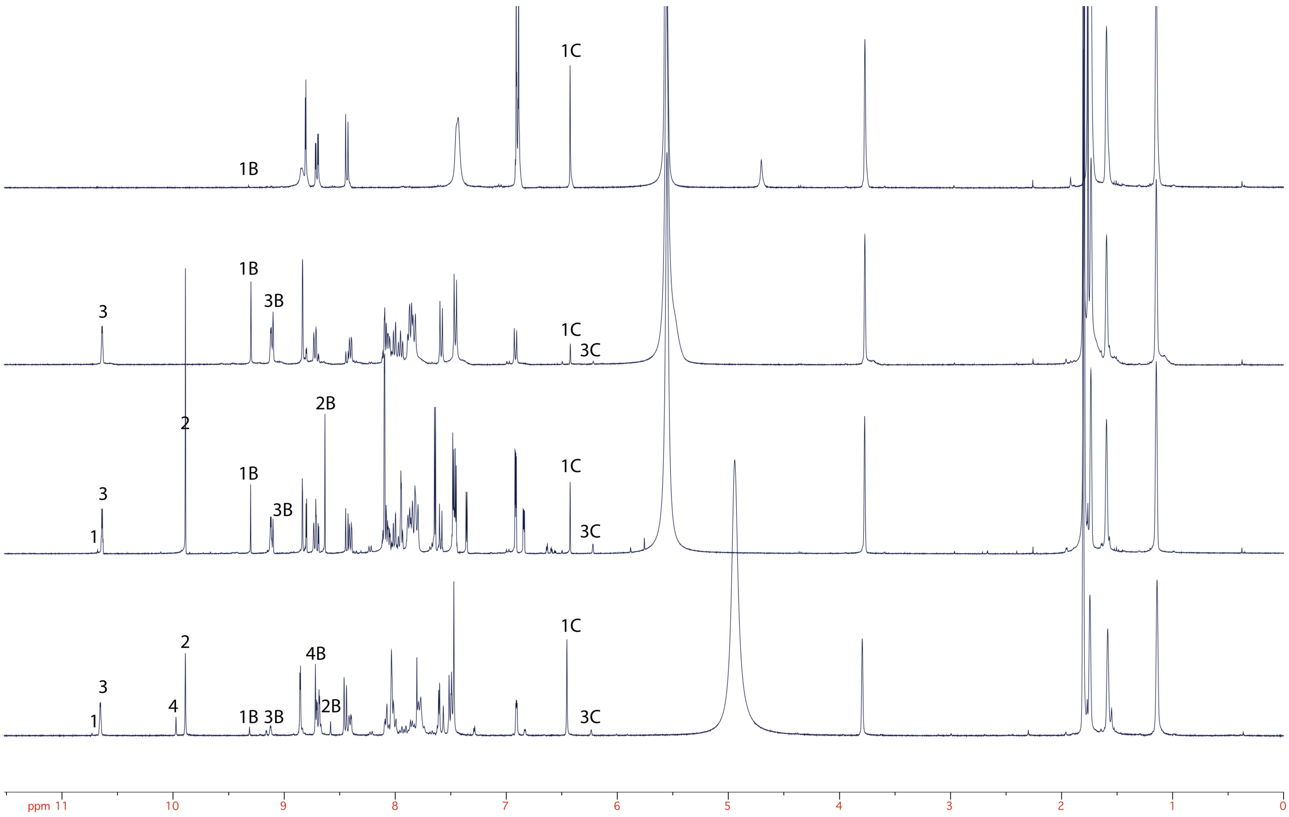

Figure S 7. ^{1}H NMR spectra of dynamic imine system **b**: 1) equilibrium of aldehyde **1**, amine **B** and deuterated *n*-butanol **C**; 2) equilibrium of aldehyde **1**, aldehyde **3**, amine **B** and deuterated *n*-butanol **C**; 3) equilibrium of aldehyde **1**, aldehyde **2**, aldehyde **3**, amine **B** and deuterated *n*-butanol **C**; 4) equilibrium of aldehyde **1**, aldehyde **2**, aldehyde **3**, aldehyde **4**, amine **B** and deuterated *n*-butanol **C**; ^{1}H NMR analyses were conducted with all building blocks of 40 mM concentration in deuterated *n*-butanol.
